# Development of individuals with thanatophoric dysplasia surviving beyond infancy

**DOI:** 10.1111/ped.15007

**Published:** 2022-02-09

**Authors:** Mariko Ushioda, Hideaki Sawai, Hironao Numabe, Gen Nishimura, Hiroaki Shibahara

**Affiliations:** ^1^ Department of Obstetrics and Gynecology Hyogo College of Medicine Nishinomiya‐shi Japan; ^2^ Department of Clinical Genetics Hyogo College of Medicine Nishinomiya‐shi Japan; ^3^ Clinical Genetics Center Tokyo Medical University Tokyo Japan; ^4^ Intractable Disease Center Saitama Medical University Hospital Moroyama Japan

**Keywords:** bioethics, genetic counseling, physical and psychosocial development, single gene disorder, thanatophoric dysplasia

## Abstract

**Background:**

This study aimed to analyze the physical and psychosocial development of long‐term survivors (age >1 year) of thanatophoric dysplasia (TD).

**Methods:**

The participants were 20 long‐term survivors recruited from a cohort obtained through a nationwide survey for TD conducted across 147 pediatric departments in Japan between 2012 and 2016. Their guardians consented to participate in this study. Medical and psychosocial information was collected through questionnaires and interviews with primary physicians and guardians.

**Results:**

The participants were 1.2–27.8 years old, and all showed marked growth deficiency. The mean length at birth was 36 cm (−3.4 SD to −7.9 SD). The adult height (age >16 years) was <−15.2 SD. All individuals showed severely delayed psychomotor development. The highest level of psychosocial development was equivalent to that at 2 years of age. Skin disorders (acanthosis nigricans and seborrheic keratoses) were common. Eleven subjects had been hospitalized or institutionalized consistently after birth, and nine had been moved to home care, and four were exclusively orally fed. All individuals required assisted ventilation.

**Conclusions:**

Long‐term survival of TD individuals is common. Some individuals enjoy home‐based lives; however, they are severely psychosocially and physically disabled and require meticulous respiratory and nutritional support.

Thanatophoric dysplasia (TD) is a severe skeletal dysplasia caused by heterozygous mutations in the gene encoding fibroblast growth factor receptor 3 (FGFR3). This disorder is the most severe of a group of FGFR3‐related skeletal dysplasias, including the prototype achondroplasia and the mild form, hypochondroplasia. The incidence of TD, including stillbirths, is reported to be 1/20 000–1/50 000. All cases, but exceptional parental germinal mosaicism, have been sporadic due to *de novo* mutations. Based on the skeletal manifestations, TD is subdivided into types 1 and 2. The overall skeletal manifestations are much more severe in type 1, whereas severe craniosynostosis is associated with type 2. Type 1 is much more prevalent. The clinical hallmarks include macrocephaly, severe shortening of the limbs, and short ribs leading to thoracic hypoplasia. Thanatophoric dysplasia individuals almost invariably develop respiratory insufficiency in the neonatal period, which usually leads to early neonatal mortality.^1^, ^2^ Recently, however, there have been a number of reports documenting the long‐term survival of TD individuals due to advances in modern neonatal respiratory care.^3^, ^4^, ^5^, ^6^, ^7^, ^8^ Previously, we assisted a research group organized by the Japanese Ministry of Health, Labour, and Welfare in conducting a nationwide survey for TD in Japan (2005–2010).^9^ The cohort included 16 long‐term survivors (over 1 year) out of 51 TD children.^9^ Thus, these studies indicate that the long‐term survival of TD infants is common. Nevertheless, very little is known about the physical and psychosocial development and quality of life in these TD survivors. The objective of this study was to clarify these issues, based on another nationwide survey of long survivors with TD.

## Methods

We have confirmed for the first time that newborns with TD may not always die during the early neonatal period but can survive the early neonatal period with appropriate respiratory management.^9^ The nationwide survey was conducted at 147 pediatrics departments (21 children's hospitals, 79 university hospitals, and 47 perinatal medical centers) between 2012 and 2016. Information about the survey was also published and disseminated through the research group's webpage.^10^ The subjects comprised 20 TD individuals who survived over 1 year, whose guardians consented to cooperate with this study. All affected individuals were diagnosed with TD type 1. The medical and psychosocial information of the subjects was collected through questionnaires and personal interviews. Survey instructions and questionnaires were mailed to the primary physicians of the affected individuals. We personally interviewed all primary physicians and, in some cases, met the affected individuals and their guardians in person. The questionnaire addressed family history, method of diagnosis, birth‐related details, postnatal respiratory and nutritional support, and motor and psychosocial development (Box 1).

**BOX 1 Individual data collected in the survey**

- Individual demographics: Age, gender, current place of care
- Family history
- Birth history: Parents' age at birth, gestational age at birth, prenatal diagnosis, delivery method, Apgar score, physical and X‐ray findings at birth
- Postnatal history: Respiratory management method, nutrition management method, genetic testing, course of physical development
- Current status: Clinical symptoms, motor development findings, psychological development findings, lifestyle

### Editorial policies and ethical considerations

Ethical permission for this study was obtained from the ethics review board of the Hyogo College of Medicine (No. 2445, 2564). The study complies with the recognized standards as required by the Declaration of Helsinki. Informed consent for publication of clinical data and photos was obtained in advance from the guardians.

## Results

### Individual demographics

The basic demographic data are summarized in Table 1. The subjects comprised 12 males and 8 females, ranging in age from 1.2–27.8 years. Fifteen individuals were aged 1–9 years, three were aged 10–19 years, and two were aged over 20 years. Eleven were receiving inpatient care, while nine were receiving home care. Home care started from 6 months to 8 years of age (average 2.2 years). The average maternal and paternal ages were 34.1 years and 34.2 years, respectively (not shown in Table 1).

**Table 1:** Summary of individual history and physical examination results

Individual (Case No.)	Age^†^	Prenatal diagnosis	Gender	GA	Method of delivery	Apgar score		Measurements at time of birth				Place of care	Genetic testing	Tracheo‐stomy	Spontaneous breathing	Respiratory care method at birth	Timing of tracheostomy	Nutritional care	Acanthosis Nigricans
						1 min	5 min	Weight (g)	Height (cm)	OFC (cm)	CC (cm)								
1	22.3	Yes	M	36 weeks 5 days	Cesarean	4	6	2,798	Unknown	Unknown	NA	Inpatient	Arg248Cys	+	‐	SIMV	8 months	Tube feeding	Generalized
2	7.6	Yes	F	40 weeks 3 days	Vaginal	8	4	2,978	38	38	NA	Home	NA	+	NA	HFO	Day 51	Oral intake (Ordinary diet)	Generalized
3	8.6	Yes (US)	M	35 weeks 2 days	Cesarean (fetal indications)	1	2	2,783	35	32.3	27.9	Home	NA	+	+	HFO	Day 131	Tube feeding	Generalized
4	2.0	Yes (CT)	M	37 weeks 5 days	Cesarean (fetal indications)	6	8	2,800	39	37.5	28.5	Inpatient	Arg248Cys	+	+	HFO	Day 97	Oral intake (Baby food)	Present
5	1.4	None	M	38 weeks 2 days	Cesarean (breech position)	2	6	2,528	37	37	26	Home	Arg248Cys	+	+	SIMV	Day 38	Tube feeding + Oral intake (Baby food)	Generalized
6	3.2	Yes (US,CT)	M	38 weeks 0 day	Vaginal	8	9	2,362	40	36	NA	Home	NA	+	+	Nasal CPAP	Day187	Tube feeding + Oral Intake (liquids/sweet)	None
7	3.25	Yes (US)	F	39 weeks 0 day	Cesarean (breech position)	4	6	2,606	32	38.2	NA	Inpatient	NA	‐	+	NA	Not performed	Tube feeding	None
8	3.2	Yes (3DCT)	M	33 weeks 5 days	Cesarean (Placental abruption)	3	8	1,720	35	32	NA	Inpatient	Tyr373Cys	‐	+	HFO	Not performed	Tube feeding	Generalized
9	5.0	Yes (US)	M	37 weeks 3 days	Cesarean	6	9	2,744	34	38.2	NA	Home	NA	+	+	HFO	3 years	Tube feeding	Forehead, axilla, vulva
10	10.0	Yes (amniotic fluid)	F	38 weeks 1 day	Cesarean (protracted labor)	5	5	3,686	41	37.8	NA	Home	Arg248Cys	+	Weak	HFO	10 months	Tube feeding	Generalized
11	6.3	Yes (US)	F	36 weeks 4 days	Vaginal	8	8	2,538	40	35	NA	Inpatient	Tyr373Cys	‐	+	NA	Not performed	Tube feeding	Forehead
12	5.6	Yes (US,CT)	F	37 weeks 2 days	Cesarean (fetal indications)	8	9	2,754	38	36.8	26.8	Inpatient	Arg248Cys	+	+	Nasal CPAP	8 months	Tube feeding	Generalized
13	13.9	Yes (US)	M	36 weeks 3 days	Cesarean (CPD)	4	6	2,464	36	37.5	27.5	Inpatient	NA^‡^	+	Weak	SIMV	3 years	Tube feeding	Generalized
14	27.8	Yes (US)	F	36 weeks 3 days	Vaginal	6	8	2,904	39	35	30.2	Inpatient	Arg248Cys	‐	+	SIMV	Not performed	Tube feeding	Generalized
15	9.4	Yes (MRI)	F	39 weeks 3 days	Cesarean (fetal arrhythmia)	3	5	3,026	37	NA	29	Home	Arg248Cys	+	Very weak	SIMV	Day 65	Oral intake (puréed food)	Generalized
16	6.7	Yes (US)	M	30 weeks 1 day	Vaginal	5	6	1,610	34	31.1	23	Home	NA	+	NA	SIMV	Day 84	Tube feeding	Forehead
17	1.2	Yes (US)	M	35 weeks 6 days	Cesarean (breech position)	7	9	2,068	34	35	25	Inpatient	Tyr373Cys	+	‐	SIMV	Day 144	Tube feeding	Neck, shoulder, buttock
18	1.8	Yes (US)	M	35 weeks 5 days	Cesarean	6	8	2,332	35	36	NA	Inpatient	NA^‡^	+	NA	CMV	Day 29	Tube Feeding	None
19	19.1	None	F	36 weeks 4 days	Cesarean (IUGR)	7	8	2,016	35	32.4	NA	Home	Translation stop codon 807Phe	+	+	NA	5 months	Oral intake (Ordinary diet)	None
20	4.25	Yes (US)	M	37 weeks 4 days	Cesarean (breech position)	6	9	2,420	38	37	NA	Inpatient	Arg248Cys	+	+	HFO	Day 268	Tube feeding + Oral Intake (Baby food)	Generalized

CC, chest circumference; CPAP, continuous positive airway pressure; CT, computerized tomography; GA, gestational age at delivery; HFO, high frequency oscillation; MRI, magnetic resonance imaging; NA, not available; OFC, occipitofrontal circumference; SIMV, synchronized intermittent mandatory ventilation; US, ultrasound; w/d, weeks/days.

^†^ Age at time of study (years).

^‡^ Molecular analysis was made, but the details were unknown.

### Prenatal, perinatal, and postnatal history

In 18/20 subjects, prenatal ultrasound diagnosis revealed severely short limbs and a large head, while two did not undergo prenatal ultrasound diagnosis. A small subset of individuals underwent other prenatal investigations using computed tomography (CT), magnetic resonance imaging (MRI), or amniotic fluid‐based analysis. A definitive prenatal diagnosis was made in only one patient who had undergone an amniotic fluid‐based molecular analysis.

Ten subjects were born prematurely, and the other ten were full term. Cesarean delivery was performed in 75% of cases for various reasons. Apgar scores at 1 min ranged from 1 to 8 (4–8 in 16/20), and Apgar scores at 5 min from 2 to 9 (6–9 in 16/20). Birth length was 36.7 ± 2.5 cm, weight 2,557 ± 475 g, occipitofrontal circumference (OFC) 33.8 ± 8.5 cm, and chest circumference 27.1 ± 2.2 cm.

### Diagnosis and genetic testing

All subjects had TD type 1 on the basis of a retrospective radiological review. Fourteen individuals underwent FGFR3 molecular analyses. Unfortunately, the molecular record was lost for two. Eight individuals had the Arg248Cys mutation, three had Tyr373Cys mutations, and one had a mutation affecting the C‐terminal end translation stop codon 807Phe.

### Postnatal physical development

**Fig. 1:**
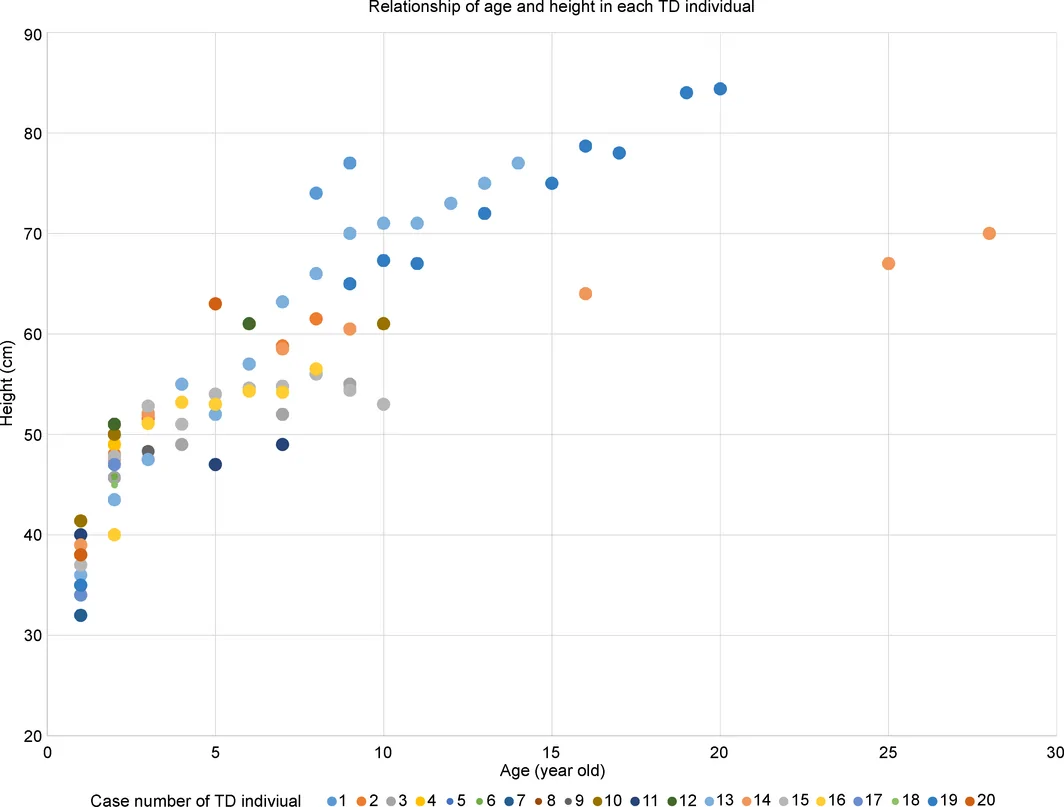
Growth chart of individuals with thanatophoric dysplasia, showing height with increasing age. Height at the age of each case is shown, with marked shortening in all cases.

The postnatal length is shown in Figure 1. At birth, the mean length was 36 cm (−3.4 SD to −7. 9 SD). From 1 to 5 years of age, length ranged from −8.9 SD to −13.8 SD; 6 to 10 years, −9.0 SD to −13.7 SD; and 11 to 15 years, −9.8 to −17.7 SD. The adult height (16 years old and up) was under −15.2 SD.^11^

### Pubertal development

A 27‐year‐old woman (individual 14) had sparse pubic hair, but she did not have axillary hair, nor did she show mammary development or menarche. A 19‐year‐old woman (individual 19) did not have menarche.

### Visual and auditory function

Visual and auditory acuity could be assessed only in some subjects. Thirteen of 17 individuals (excluding individuals 5, 17, and 18 who passed away in early childhood) were able to move their eyes to follow a moving object (capability of visual tracking). Eleven of 16 evaluable individuals responded to auditory stimuli by looking at the source of a sound. One individual wore a hearing aid due to severe hearing impairment.

### Neuroimaging and neurophysiological findings

Neuroimaging was available in 12 subjects, all of whom had hydrocephalus. Nine showed severe stenosis of the foramen magnum. Three individuals underwent occipital decompression surgery, and one each underwent VP shunt procedure and Ommaya reservoir placement. Sixteen patients had convulsive seizures that were treatable with standard antiepileptic regimens.

### Gastrointestinal, urinary, and autonomic dysfunction

All subjects showed marked abdominal distension, mainly related to respiratory support. Seventeen of 20 individuals experienced severe constipation, requiring laxatives. Only two patients were able to control bowel movements on their own. Three individuals had difficulty in urination, requiring urethral catheterization and/or hypogastric compression to stimulate voiding. All individuals had impairment of body temperature control, which frequently led to fever without any signs of infection. Three individuals experienced intermittent bradycardia that occurred primarily during sleep.

### Dermatological findings

**Fig. 2:**
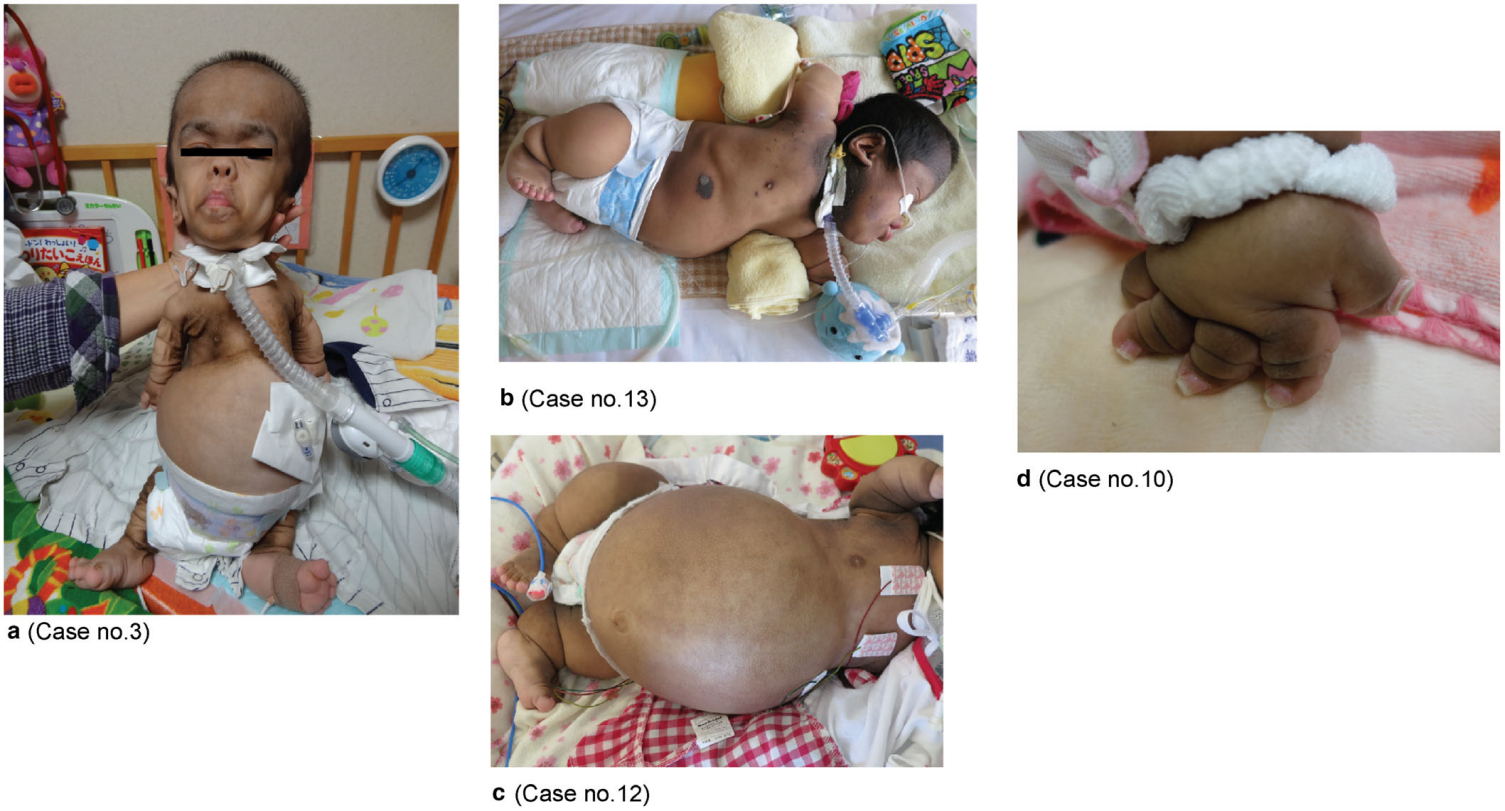
Dermatological findings of acanthosis nigricans. Individuals with acanthosis nigricans are shown, predominantly affected in (a) the chest region in individual 3 (b) the forehead and cervical region in individual 13, (c) the axillary region in individual 12, and (d) the hand in individual 10.

**Fig. 3:**
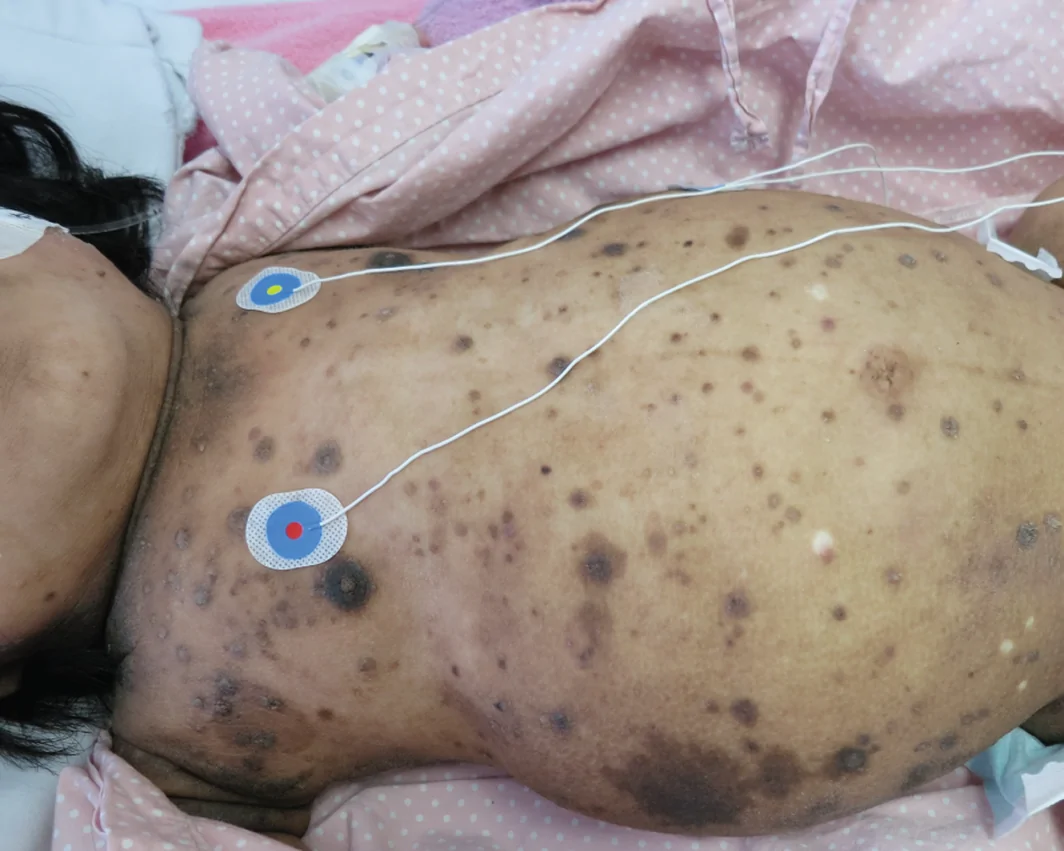
Dermatological findings of seborrheic keratosis. Seborrheic keratosis over the anterior chest and abdominal wall (individual 14) is shown.

Acanthosis nigricans was observed in 16 individuals (Fig. 2). Brown hyperpigmentation deteriorated with age. The forehead and flexural regions (neck, axilla, and joints) were most commonly affected. In addition, three individuals had verruca, which was pathologically diagnosed with seborrheic keratoses in one individual **(**Fig. 3).

### Motor, cognitive, and psychosocial development

**Fig. 4:**
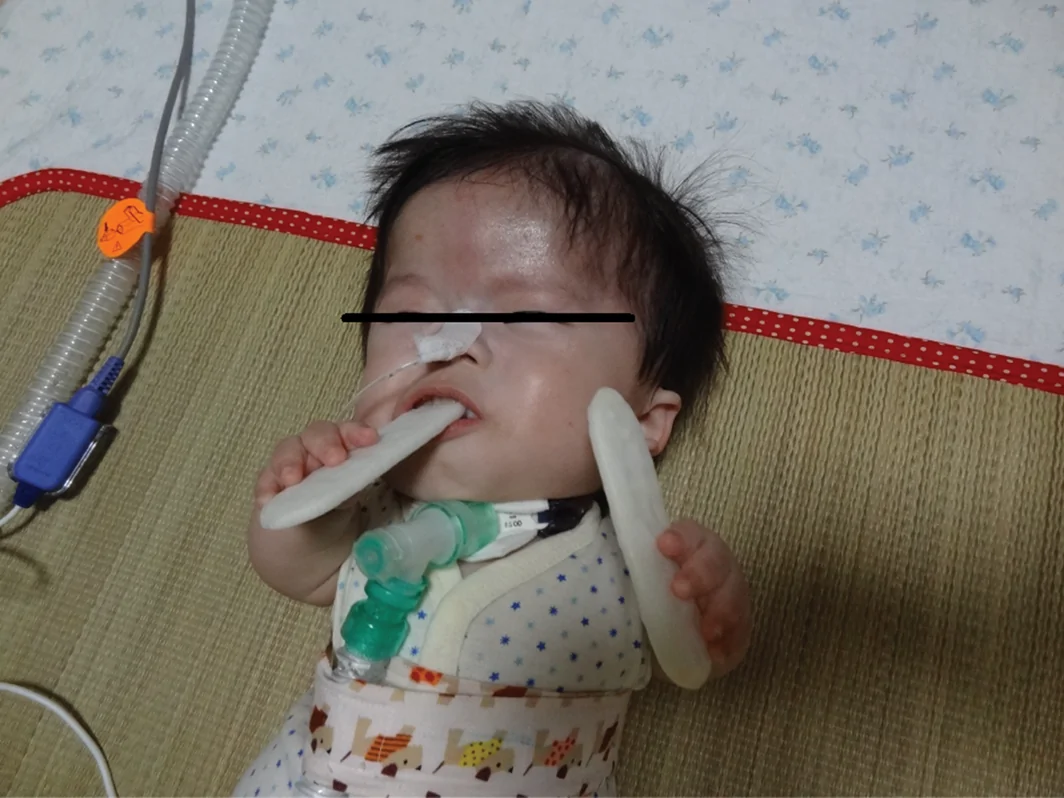
Motor function. An individual with favorable motor function demonstrates grasping a candy and bringing it to his mouth (individual 6).

None of the subjects was able to rise from the floor, stand, or walk alone. Thirteen individuals had marginal to modest limb movements. A few individuals were able to point with a finger, bring their hands to their face, slither across the floor on the back, turn on the side, and roll over. Individuals with the highest level of development were able to hold a piece of candy and bring it to their mouth, hit a tambourine, and give a high five (Fig. 4). All individuals winced or cried when hurt, while most (19 of 20 individuals) laughed when cuddled. Psychosocial development could be examined in only 15 subjects. Ten of them were shy towards strangers. The psychosocial development of each individual whom we met in person is detailed in Table S1. Psychosocial development did not occur over 3 months in seven out of 20 individuals. The highest level corresponded to that of 2 years of age.

### Respiratory support and care

All affected individuals required varying degrees of respiratory support, assisted by a ventilator. In the neonatal period, most individuals required mechanical ventilation with intratracheal intubation soon after birth. Two individuals needed only nasal CPAP: one for about 11 months (individual 6), and the other 3 months (individual 12) after birth. One patient (individual 13) was extubated shortly after initial intubation; however, he developed respiratory failure necessitating reintubation. Sixteen individuals ultimately underwent tracheotomy, and 14 underwent tracheotomy within 1 year after birth. Tracheostomy was not attempted in four individuals who were considered to be at high risk of surgical complications (a very short, wide neck is susceptible to tracheo‐innominate artery fistula). The current respiratory status varied among individuals. Individual 6 had sleep apnea requiring different respiratory support setups between day and night‐time. Individual 12 was respirator‐dependent with different settings between day and night‐time. Individual 19 did not need ventilator assistance during the daytime but required intermittent oxygen administration when necessary. Individual 9 required only ventilation support with indoor air when the respiratory condition was stable.

### Nutritional care

Thirteen of the 20 subjects were solely tube fed, three were tube and orally fed in combination, and four were exclusively orally fed. Fourteen individuals were tube fed via a nasogastric tube and two via a gastrostomy tube. Enrichment of oral ingestion was usually not attempted in the first group but some individuals enjoyed tasting new things; for example, eating soup or licking ice cream. Individuals in the second group were either reliant on tube feeding (oral intake was introduced for swallow training and enrichment) or oral feeding (tube feeding was used as a supplement when oral intake was difficult). Some went back and forth between the first and second groups. In the second and third groups, food used for oral intake comprised baby formula and children's food, including Japanese rice crackers, puréed food, and ordinary food. Some individuals showed a preference for certain flavors. One had repeated episodes of pneumonia that led to laryngotracheal separation but was currently being orally fed with puréed food. Individuals in the third group showed almost normal dental development, and even those in the first and second groups showed only a mild delay in dentition.

## Discussion

### General

In this nationwide survey for long‐term survivors with TD (type 1), we compiled data on physical, motor, and psychosocial development in 20 affected individuals. All cases were sporadic. Prenatal ultrasonography raised suspicion of severe bone dysplasia in most affected individuals (18/20). In individuals who had a molecular diagnosis (14/20), Arg248Cys and Tyr373Cys mutations were common, as previously reported. The increase in body length was constant during childhood. However, as is common in skeletal dysplasia, the pubertal growth spurt was absent. The adult length (70–84 cm) was identical to that previously reported.^6^ Visual and auditory functions were generally well preserved but they might have been impaired by neurological complications. In fact, stenosis of the foramen magnum and hydrocephalus are essential syndromic components of TD, which may necessitate neurosurgical intervention. Seizures were also common, but could be treated using standard antiepileptic therapies. Some individuals had gastrointestinal and urinary difficulties. Mild autonomic instability may be common.

### Skin

Distinctive dermopathy is another essential syndromic component of TD. Many affected individuals had acanthosis nigricans. It is known that acanthosis nigricans is associated with other FGFR3‐related skeletal dysplasias; however, dermopathy is prevalent and severe in TD.^12^ A few individuals developed seborrheic keratosis, which commonly occurs in older individuals after prolonged exposure to UV light.^13^ It is intriguing that senile dermopathy is associated with the *FGFR3* Arg248Cys somatic mutation.^5^, ^14^ Germinal mutation of Arg248Cys is responsible for early onset with minimal exposure to sunlight of seborrheic keratosis in TD.

### Motor, cognitive, psychosocial development

All individuals showed severe retardation of motor, cognitive, and psychosocial development. The highest level of psychosocial development did not go beyond 2 years. Hydrocephalus and stenosis of the foramen magnum may be partly responsible for this developmental delay, particularly for motor delay.^8^, ^15^ However, generalized developmental delay is essentially attributed to brain malformation.^16^, ^17^

### Management

Respiratory and nutritional support is of utmost importance in the management of TD individuals. As has been reported previously, most individuals require continuous assisted ventilation; however, some required only intermittent support.^13^ Most individuals required tracheostomy for respiratory support. Respiratory failure in TD is attributed to pulmonary hypoplasia secondary to thoracic cage hypoplasia. Brain stem dysfunction due to stenosis of the foramen magnum may also, in part, be responsible for respiratory difficulties, particularly the central apnea.^18^, ^19^ Foramen magnum decompression has been reported to improve respiratory status in some TD individuals.^8^ In our cohort, however, nobody who underwent the procedure was weaned off from assisted ventilation.

The majority of affected individuals required tube feeding. Only four individuals required oral feeding exclusively, while three had a combination of tube and oral feeding. Difficulty in oral feeding was probably related to delayed development of oro‐pharyngeal and swallowing function, and may have been in part related to long‐term intubation. Notably, dental development was normal in all individuals. Some individuals enjoyed eating, as previously reported.^15^ However, others were reluctant to take food orally, probably due to discomfort during swallowing. Thus, feeding care should be carefully individualized.

Secure respiratory and nutritional care can be facilitated by home care instead of institutionalized care. In our cohort, nine of 20 individuals were cared for at home. Transition from inpatient care to home care was feasible at an average age of 2.2 years, consistent with a previous report of an affected child who was transferred to home care at 1.5 years of age.^6^ Home care is expected to have a positive effect on psychosocial development. Needless to say, meticulous monitoring of respiratory status, skilled airway cleaning, and prompt adjustment of respiratory settings guarantees safe home care. Caregivers and family members should be well trained in these issues. Home care may impose a burden on family members. Therefore, empathetic counseling and psychological support should also be continued.

The accuracy and comprehensiveness of the study are limited because the study was conducted by questionnaire and interview with a limited number of doctors and parents and patients.

### Conclusions

It is predicted that the long‐term survival of TD individuals has increased as medical management has become sophisticated. In all individuals, however, continuous respiratory and physical care was required. Affected individuals are severely disabled psychosocially and physically, but they can enjoy home life if meticulous respiratory and nutritional care are provided.

## Disclosure

The authors declare no conflict of interest.

## Funding information

This work was supported by Research on Rare and Intractable Diseases, Health and Labour Sciences research grants H22 nanchi‐ippan‐046, H23 nanchi‐ippan‐123, H26 nanchitou(nan)‐ippan‐055, H28‐nanchitou(nan)‐ippan‐017, and 19FC1006.

## Author contributions

M.U. designed the study, performed the study, obtained general results and wrote the manuscript. H.S. supervised this study and wrote part of the manuscript. H.N. helped in the conduct of the study. G.N. provided conceptual advice on thanatophoric dysplasia and wrote part of the manuscript. H.S. managed the conduct of this study. All authors read and approved the final manuscript.

## Supporting information


**Tab S1.** Characteristics of individuals' development.

## Data Availability

The data supporting the findings of this study are available on request from the corresponding author. The data are not publicly available due to privacy or ethical restrictions.
