# Supplementary material for: Development of individuals with thanatophoric dysplasia surviving beyond infancy
Source: Pediatr Int. 2022 Feb 9;64(1):e15007. doi: 10.1111/ped.15007 (PMC9305791; doi:10.1111/ped.15007)
Supplement: Supplementary file 1 — Tab S1. Characteristics of individuals' development. [file PED-64-0-s001.docx]

**Supplemental Table 1.** Characteristics of individuals’ development

| Individual number | Psychosocial development |
| --- | --- |
| 2 | Shows understanding of simple instructions (e.g. Please wait etc) and also exhibits patience. |
|  | Expresses the desire （when hungry）by saying "Aa-Aa-". |
|  | Plays tambourine in sync with the music. |
| 4 | Conveys his will/intention through gestures. |
|  | Understands simple instructions. |
| 5 | When upset, brushes off with hand. |
|  | Rings a bell when he wants to call someone. |
| 6 | Conveys his wish/intention（When he wants his mother to come to him, he deliberately rings the alarm, which generally goes off when the tube comes off.） |
|  | When he sees another person dancing, he imitates them and dances by shaking his body. |
|  | When he feels sleepy, he has the habit of putting gauze on his face. |
| 10 | Follows the pictures in the picture book with her eyes. |
|  | Follows the images in the DVD with her eyes. |
|  | Opens her mouth when she sees the tooth brush. |
|  | Does not follow instructions when she is upset. |
|  | Smiles when she eats her favorite food. |
|  | Plays with the feeding tube with her tongue. |
| 12 | Tries to express her feelings vocally. |
|  | While calling people, she moves her neck and deliberately removes the respiratory tube. |
|  | Dislikes when someone goes away and conveys this feeling with voice or expressions. |
|  | Gives a high-five when she is in a good mood. |
|  | Shakes her head and dances to the tunes of her favorite music. |
| 13 | When nobody is around, he calls. |
|  | To call people, he makes efforts to ring the alarm by bringing SpO_2_ (level) down. |
|  | Understands basic instructions（when asked to wait, he will become quiet.） |
|  | Puts his tongue out when upset. |
|  | Conveys his wish/intention by gestures.（blinking） |
| 15 | Plays by shaking the toy fastened to her wrist. |
|  | When her name is being called out, she looks in the direction of the voice. |
|  | Opens her mouth when she sees juice. |
| 19 | Understands simple instructions and rules. |
|  | Expresses her feelings by moving her neck. |
|  | Understands the feelings of the other person. |
|  | Expresses her feelings by using sounds like "Aa-Aa-". |
|  | Puts her hands on ears when there is noise. |
|  | She is picky about food. |
| 20 | Understands simple instructions.（Opens the mouth as "Aaaa-" when eating food.） |
|  | Expresses his feelings with facial expressions. |
